# A multi-agent GraphRAG framework for pharmacotherapy safety verification in clinical decision support systems

**DOI:** 10.3389/fmed.2026.1898857

**Published:** 2026-08-26

**Authors:** Victor Ryzhenko, Bogdan Burlaka, Igor Belenichev, Kristina Burlaka, Olena Aliyeva, Nina Bukhtiyarova, Iryna Halabitska, Pavlo Petakh, Oleksandr Kamyshnyi

**Affiliations:** 1 Department of Medical and Pharmaceutical Informatics and Advanced Technologies, Zaporizhzhia State Medical and Pharmaceutical University, Zaporizhzhia, Ukraine; 2 Department of Medicines Technology, Zaporizhzhia State Medical and Pharmaceutical University, Zaporizhzhia, Ukraine; 3 Department of Pharmacology and Medical Formulation with Course of Normal Physiology, Zaporizhzhia State Medical and Pharmaceutical University, Zaporizhzhia, Ukraine; 4 Department of Clinical Laboratory Diagnostics, Zaporizhzhia State Medical and Pharmaceutical University, Zaporizhzhia, Ukraine; 5 Department of Histology, Cytology and Embryology, Zaporizhzhia State Medical and Pharmaceutical University, Zaporizhzhia, Ukraine; 6 Department of Therapy and Family Medicine, I. Horbachevsky Ternopil National Medical University, Ternopil, Ukraine; 7 Department of Biochemistry and Pharmacology, Uzhhorod National University, Uzhhorod, Ukraine; 8 Kyiv School of Economics, Kyiv, Ukraine; 9 Department of Microbiology, Virology, and Immunology, I. Horbachevsky Ternopil National Medical University, Ternopil, Ukraine

**Keywords:** CDSS, deep learning, graph neural networks, GraphRAG, multi-agent systems, precision medicine

## Abstract

**Background:**

The management of pharmacotherapy in patients with multimorbidity and polypharmacy is a difficult task in routine clinical practice. Clinicians must consider diagnoses, prescribed drugs, contraindications, drug-drug interactions, renal and hepatic function, laboratory values, dose limits and individual risk factors. Clinical decision support systems in traditional settings are based on static rules, and hard to update. Standalone large language models are capable of processing clinical language but their outputs may be unsupported by evidence, incomplete or out of date. In this study, we developed and evaluated a hybrid multi-agent GraphRAG framework for personalised pharmacotherapy safety verification.

**Methods:**

We developed a system that integrates graph based retrieval with large language models. The framework was composed of three parts: (1) a schema for a pharmacotherapy knowledge graph that encoded indications, contraindications, interactions, laboratory thresholds, dose limitations and relevant patient conditions; (2) an Extractor–Critic workflow that transformed unstructured medical text into graph elements with quality checks prior to their incorporation; and (3) a retrieval and re-ranking module that leveraged semantic search, graph traversal and safety-directed scoring. The system was evaluated on 12 benchmark cases extraction and 30 clinical safety questions answering. Comparison of conventional RAG and GraphRAG was done using linear mixed effects models, permutation testing, and paired case-level analysis.

**Results:**

The multi-agent extraction workflow beat the zero-shot baseline. Strict F1 improved by 0.156 points (*p* < 0.01), mainly due to higher ecall (+0.172, *p* < 0.001). For clinical question answering, we found that GraphRAG outperformed conventional RAG in terms of medical accuracy (0.545 vs. 0.443, absolute difference of 0.102, *p* = 0.04). GraphRAG provided the better answer 66.7% of the time. The advantage was greatest where safety was dependent on contra-indications, laboratory cut-offs or patient specific factors. Smaller models performed as well as the larger models with the same GraphRAG configuration.

**Conclusion:**

The evaluation revealed that the integration of LLM-based extraction with a pharmacotherapy knowledge graph enhanced safety-oriented clinical decision support. GraphRAG provided more accurate and traceable answers than traditional RAG. Routine adoption will require validation on larger graphs, a wider range of datasets and real clinical populations, but this approach may be appropriate for cost sensitive and privacy conscious workflows.

## 1 Introduction

The rapid evolution of evidence-based medicine and the exponential growth of biomedical data volumes have created an unprecedented cognitive burden on clinicians (1–3). As the number of polymorbidity and polypharmacy rises, the prescription of drugs has moved from a linear drug choice to a multifactorial optimisation problem that needs to take into account thousands of possible drug interactions, genetic factors, and contraindications specific to each patient. Therefore, combining medications—aimed either at multiple targets simultaneously or at enhancing the effect on a single target—may represent a significant reserve for improving therapy across various pathologies (4–7). The use of information technologies to predict the efficacy and safety of possible drug combinations, including those from different pharmacological groups, is considered promising. Modern humanity has accumulated an enormous volume of medical knowledge, which, unfortunately, is not fully utilized in clinical practice. Processing these data goes beyond the capabilities of the human brain; it is simply impossible to retain such an amount of information mentally (8, 9). Moreover, decisions often have to be made under time pressure, while also considering the high cost of each error (since patient treatment is involved), making the provision of clinicians with modern decision-support tools increasingly urgent. Therefore, current emphasis is placed on informational support for physicians and on creating a new informational environment for their work, as this is precisely what enables improvement in the quality and accessibility of treatment. However, modern technologies for substantiating drug combinations are primarily aimed only at identifying potential adverse reactions and include the use of automated prescription checks (10–12). Thus, traditional methods of clinical verification, relying on physician memory or static reference materials, are becoming sources of medical errors, which underscores the need for next-generation automated clinical decision support systems (13–16).

The first generation of clinical decision support systems was based on expert systems with rigid rule-based approaches. Although such systems ensured high accuracy in standard scenarios, they demonstrated critical inflexibility when working with unstructured data from real clinical practice. The main limitation was the scaling of input data. The development and maintenance of such systems required the formation of costly interdisciplinary teams consisting of clinical experts and specialized knowledge engineers. The latter acted as intermediaries who had to “translate” the physician's intuitive experience into the formal language of IF-THEN rules. This process made system maintenance extremely resource-intensive. Any update of a medical protocol required not simply uploading a new file, but repeated work by engineers to refactor conflicting rules, which made system scaling economically unviable (17, 18).

Prior to the emergence of generative models, information extraction from medical texts relied on fragmented pipelines of named entity recognition (NER). These systems (based on CRF, BiLSTM, or early BERT) (19, 20) required the creation of massive manually annotated data corpora for each type of entity separately (separate models for drugs, separate ones for diagnoses). Moreover, traditional NER operated in a purely “syntactic” manner: it could identify the name of a drug in text but was often unable to recognize the context of negation (“the patient is not taking…”) or temporal scope (“previously took…”) (21, 22).

The modern development of large language models (LLMs), based on transformer architecture, and has shifted the paradigm from recognition to understanding with generalization. Due to zero-shot and few-shot learning mechanisms, modern models do not require specialized fine-tuning to extract new types of data (23, 24). They can not only tag terms, but can also extract relations in one pass, correctly interpreting complex linguistic constructions and negations and causal relations that classical algorithms were not able to. However, the stochastic nature of generative models creates a fundamental problem for medicine—the tendency toward “hallucinations” (confabulations), where the model generates plausible but factually incorrect statements. In pharmacotherapy, the lack of consensus on facts makes “pure” LLMs unsuitable for important decisions (25, 26).

Another fundamental limitation of LLMs is the static nature of their memory. A model's knowledge is limited by the date its training was completed. In the fast-paced field of medicine, where treatment protocols and drug safety information (such as product recalls or new FDA data) are updated daily, using a “frozen” model poses a significant risk. At the same time, continuously fine-tuning the model is an economically unfeasible and slow process.

Retrieval-Augmented Generation (RAG) technology partially addresses this issue by dividing the process into two components: “thinking” (LLM) and “knowledge” (external database). The RAG architecture allows the model to access up-to-date external sources in real time without changing the neural network's weights, enabling this knowledge base to be used in the future for developing intelligent agents (27–29).

In clinical practice, it is not only the correct answer that matters, but also the reference to the source. A classic LLM generates text based on word probabilities, without any understanding of the source of the information. RAG changes this pattern: the generation of the answer is based exclusively on relevant fragments (chunks) extracted from verified medical documents. This allows the physician to verify each recommendation by reviewing the scientific article, original protocol, or guideline referenced by the system (30–32).

Despite their high performance in semantic analysis, LLMs make significant errors when performing arithmetic calculations. This is due to their architecture: LLMs function as probabilistic predictors of the next token, rather than as logical computing machines. In pharmacotherapy, where dosing often depends on a patient's weight, age, or kidney function, such “arithmetic hallucinations” pose a critical risk. Even the most powerful LLMs can make mistakes in simple calculations or confuse units of measurement, which is unacceptable in clinical practice (33, 34).

Given the above, the design and development of a hybrid multi-agent architecture for personalized medicine systems is a relevant and promising direction.

**The aim of this work** is to develop and justify a hybrid multi-agent architecture for personalized medicine systems that combines deep learning methods and GraphRAG technology to predict therapeutic strategies and automate the verification of drug prescription safety, while minimizing AI hallucinations and ensuring the explainability of the decisions made.

Our Proposed Method: **Agentic Graph-Guided RAG Architecture.**

**Figure 1:**
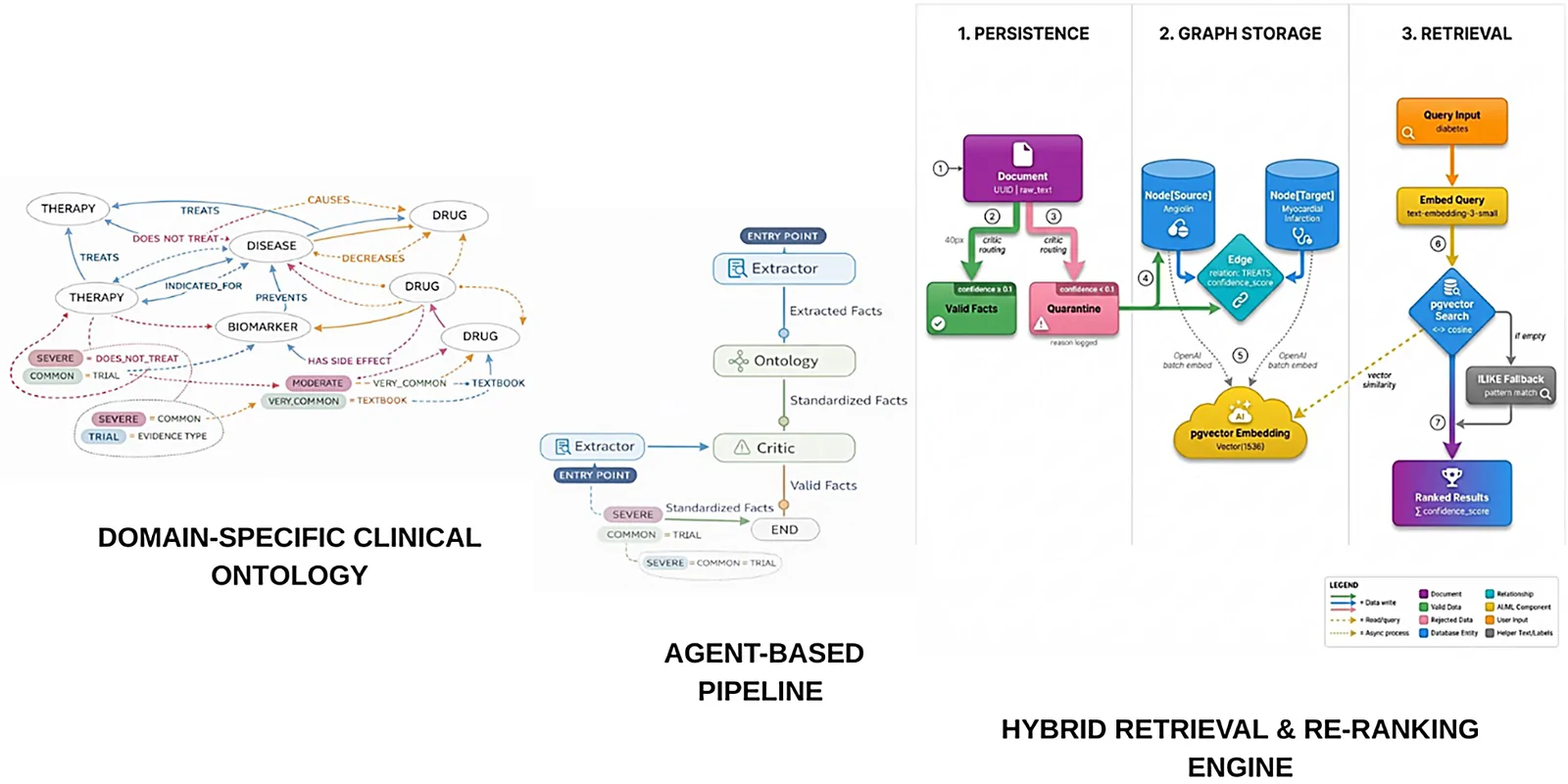
Agentic graph-guided RAG architecture.

We propose architecture (https://github.com/burlakabogdan/PharmaRec_public) based on the following key components: (1) a clinical ontology optimized for logical inference, (2) an agentic pipeline for graph completion, and (3) a hybrid module for information retrieval and adaptive ranking (Figure 1).

*1. Clinical Ontology (Ontology-inspired domain-specific knowledge graph schema).* Unlike large universal ontologies, such as SNOMED CT (35), which contains millions of concepts but is difficult to navigate, our ontology schema is designed to support active decision-making. It is based on a directed graph where edges are typed by functional impact. The choice of relation types (RelationType) is driven by the following requirements:

A. Explicit Negation for Safety. Standard RAG systems often ignore the word “not,” confusing “treats” and “does not treat.” We introduced the edges DOES_NOT_TREAT and NOT_INDICATED_FOR. This allowed the graph technology to instantly cut off invalid paths. Example: Antibiotics-[DOES_NOT_TREAT]->Viral Infection. This created a “hard barrier” (Hard Negative) for the system, preventing hallucinations.

B. Quantitative Reasoning Support. A significant number of medical errors are linked to the incorrect interpretation of laboratory test results. Instead of the general “Affects” relationship, the vectors INCREASES and DECREASES were created. This allowed the system to perform checks such as: If Patient. K > 5.5 AND Drug-[INCREASES]->Potassium, TO Warning. Without this distinction, automatic verification of test results would be complicated.

C. Metabolic and Interaction Logic. To detect non-obvious drug interactions, the relationships INHIBITS and METABOLIZED_BY were introduced. This allowed us to identify interaction chains: Drug A-[INHIBITS]->CYP3A4 < -[METABOLIZED_BY]-Drug B. The direct text of the instructions may not mention the conflict between A and B, but the graph deductively concludes this.

D. Granularity of Attributes. In addition to the relationship type, each edge was enriched with the attributes IntensityLevel (MILD, SEVERE) and Frequency (RARE, COMMON). This allowed us to prioritize risks: a “rare but lethal” side effect carries more weight than a “frequent but mild” one.

*2. Agent-based pipeline.* To convert unstructured text (e.g., medication instructions, scientific articles, protocols) into the ontology described above, we have added an agent-based process:

Phase 1: The Extractor Agent. This agent is responsible for the initial analysis of the text and the identification of entities (NER) and relationships (RE). Entity Normalization (Cross-Lingual Normalization): The agent accepts input in Ukrainian or English but normalizes entity names to their canonical English forms (e.g., “Гідазепам” = “Gidazepam,” “Ниркова недостатність” = “Kidney Failure”). This solves the language barrier problem during search. Context Awareness: the agent analyzes the context of a sentence to determine conditional relationships (e.g., CONTRAINDICATED_WITH only if eGFR < 30).

Phase 2: The Critic Agent does not generate new facts but acts as a quality engineer. It checks the extractor agent's output against strict rules:

- Schema Enforcement: Does the detected relation belong to the allowed list of RelationTypes? If the extractor agent invented the relation “IS_BAD_FOR,” the Critic Agent rejects it.
- Negation Check: the critic agent re-reads the sentence to ensure that the extractor agent did not overlook a negation (for example, did not convert “does not affect blood pressure” into “affects blood pressure”).
- Entity Resolution: The critic agent checks whether the found concept is a valid medical entity rather than a generic word (for example, it removes triples of the type Drug->TREATS->“Symptoms” as too abstract).

Only facts that have been verified by a reviewer (“Valid Facts”) are included in the final Knowledge Graph. Facts of questionable quality (based on confidence settings) are sent to “Quarantine” (quarantine_facts) for manual review.

*3. Hybrid Retrieval and Re-ranking Engine.* In the first stage, the system transforms the input set of clinical conditions (symptoms/conditions) C = {c1, c2, … } into a vector space. We use an embedding model (OpenAI, text-embedding-3-small) through which each condition ci is converted into a vector $V_{ci}\in \;R^{n}$. The search for potential therapeutic targets (Disease Nodes) in the Knowledge Graph is performed by calculating cosine similarity:

$$\mathrm{Target}(c_{i})=\arg\;\max_{t\in G}\left(\frac{v_{c_{i}}\cdot v_{t}}{\left|v_{c_{i}}||v_{t}\right|}\right)$$

This allowed the system to identify relevant nosologies even in the face of terminological discrepancies (e.g., “renal insufficiency” or “renal failure”). For the identified targets, the system initiates a graph traversal procedure to identify candidate drugs. A hybrid search mode (Hybrid Scope Mode) is used:

- local search (Doc-Scoped). It is limited to graph edges derived from specific clinical data (scientific articles, protocols, text data);
- global search (Global Fallback). If local data is insufficient, the search is expanded to the entire graph.

The base candidate score Sbase(d) is calculated as the sum of the confidence scores of edges of type TREATS:

$$S_{\mathrm{base}}(d)=\underset{e\in E_{\mathrm{treats}}}{\sum }\mathrm{conf}(e)$$

Penalty mechanism for drug contraindications (Safety Penalty Function). To ensure safety, a “soft constraints” algorithm is used. The system scans edges of the type CONTRAINDICATED_WITH between candidate *d* and other patient states. Identified risks are implemented through a penalty function with a coefficient of *α* = 2.0 (penalties have double the weight compared to benefits, which corresponds to the principle of *primum non nocere*):

$$S_{\mathrm{safe}}(d)=\underset{\mathrm{Efficacy}}{\underbrace{\overset{N}{\underset{i=1}{\sum }}C(e_{\mathrm{treats}}^{\left(i\right)})}}-\alpha \cdot \underset{\mathrm{Risk}\;\mathrm{Penalty}}{\underbrace{\overset{M}{\underset{\;j=1}{\sum }}C(e_{\mathrm{contra}}^{\left(j\right)})}}$$

S_safe_(d)—Safety-Adjusted Base Score.

This component reflects the overall clinical utility of the drug according to the knowledge graph, adjusted for contraindications.

$\sum e_{\mathrm{treats}}$ (Efficacy): The sum of the confidence scores of all edges of type TREATS in the graph that link drug d to the patient's diagnosed conditions. Reflects the strength of the evidence base for efficacy.

$\sum e_{\mathrm{contra}}$ (Risk): The sum of the confidence scores of the CONTRAINDICATED_WITH edges linking the drug to the patient's comorbidities.

***α*** (Penalty Coefficient): The risk penalty coefficient. Value in the source code: ***α*** = 2.0. In accordance with the principle of “primum non nocere” (do no harm), a safety signal carries twice the weight of an efficacy signal. This ensures that an effective but dangerous drug will rank lower in the recommendations than a less effective but safe one.

Pharmacodynamic evaluation $\Delta_{\mathrm{lab}}\;$ (Lab Gating and Re-ranking). The system analyzes the vectors of the drug's effect on biomarkers (INCREASES/DECREASES) and compares them with the patient's deviations (LAB_HIGH/LAB_LOW). The $\Delta_{\mathrm{lab}}\;$ weight correction is calculated using the formula:

$$\Delta_{\mathrm{lab}}=\overset{K}{\underset{k=1}{\sum }}(w_{k}\cdot C(e_{\mathrm{interact}}^{\left(k\right)})\cdot \mathrm{sgn}(\mathrm{Ef}f_{k}))$$

where:

$e_{\mathrm{interact}}^{\left(k\right)}$—an edge in the graph of type INCREASES or DECREASES connecting drug d with biomarker k (for example, “Drug A->INCREASES->Creatinine”).

$C(e_{\mathrm{interact}}^{\left(k\right)})$—the confidence coefficient of this edge (how confident the system is in this effect).

$w_{k}$—Biomarker Criticality Weight. Reflects the clinical importance of a specific biomarker.

$\mathrm{sgn}(\mathrm{Ef}f_{k})$—Direction Vector: determines whether the effect is beneficial (+1) or harmful (−1).

+1: the drug lowers a parameter that is high in the patient. The drug raises a parameter that is low in the patient.

−1: The drug increases an already high indicator (exacerbating the condition). The drug lowers an already low indicator.

Final ranking formula:

$$S_{\mathrm{final}}(d)=S_{\mathrm{safe}}(d)+\Delta_{\mathrm{lab}}$$

where:

*S*_final_(*d*) is the Aggregate Suitability Score.

This is the final numerical value used to rank candidate drugs *d* in the list of recommendations. The higher the value, the more suitable the drug is for the patient in terms of the “efficacy/safety/personalization” balance. Range of values: (−∞, +∞). Negative values indicate that risks outweigh benefits.

Here is an example of calculations based on our proposed final ranking formula:

Suppose we are evaluating the drug ibuprofen (*d*).

1. Efficacy (S_base_): treats “Headache” (conf = 0.9) and “Fever” (conf = 0.8).
  $$S_{\mathrm{base}}=0,9+0,8=1,7$$
2. Safety (S_contra_). The patient has gastritis. The Knowledge Graph shows CONTRAINDICATED_WITH (conf = 0.6).
  $$S_{\mathrm{safe}}=1,7-(2,0*0,6)=0,5$$
3. Laboratory values $\Delta_{\mathrm{lab}}$:
  - The patient has HIGH creatinine (Critical, w = 3.0).
  - Knowledge graph: ibuprofen->INCREASES->creatinine (conf = 0.7).
  - This is harmful (−1).
    $$\Delta_{\mathrm{lab}}=3,0*0,7*(-1)=-2,1$$
4. Final result:
  $$S_{\mathrm{final}}=0,5+(-2,1)=-1,6$$

Based on the final result, the system concludes: the drug has a negative rating and will be hidden or displayed at the bottom of the list with a warning, despite the fact that it is effective against headaches.

## 2 Material and methods

The platform was developed using the Python programming language (v3.13) and the following LLM orchestration frameworks: LangChain (v1.1.3) for agent orchestration and prompt templating; LangGraph (v1.0.4) for executing multi-agent work processes with conditional routing; LangChain Core (v1.1.3)—basic abstractions for interacting with LLMs; PostgreSQL database and vector store (psycopg2-binary v2.9.11)—a relational store for the knowledge graph, pgvector (v0.4.2)—extension for similarity-based vector search for semantic retrieval, SQLAlchemy(v2.0.45)—ORM layer with type-safe schema definitions, Alembic(v1.17.2)—database migration management; semantic embeddings—Sentence-Transformers (v5.2.0)—support for local embedding models for evaluation benchmarks, OpenAI text-embedding-3-small (1,536 dimensions)—production embedding model; web service framework: FastAPI (v0.124.0)—implementation of a REST API with automatic generation of OpenAPI documentation; frontend framework: React (v19.2.0); evaluation and statistical analysis: DeepEval (v3.7.5)—a framework for LLM-based evaluation metrics, Pingouin (v0.5.5)—statistical hypothesis testing for cross-model comparisons; Statsmodels (v0.14.6)—advanced statistical modeling; Matplotlib (v3.10.7), Seaborn (v0.13.2)—visualization.

### 2.1 Data collection and dataset description

To comprehensively evaluate the proposed architecture, we developed a two-stage validation strategy that includes two specialized test datasets (Dataset A, Dataset B). By using this method, we can separate between errors that happen during the knowledge extraction phase and errors that happen during the clinical decision making phase.

*Dataset A:* Extraction Benchmark (8_extraction_dataset.json). This dataset is used to evaluate the system's ability to convert unstructured medical text into a structured Knowledge Graph. Scope: 12 test cases covering complex linguistic constructions. Test categories in Dataset A:

- Simple facts (Direct Extraction)—example: “Drug A treats Disease B”;
- Negations (Hard Negatives)—example: “Antibiotics do not work against viruses”;
- Cross-lingual Normalization—example: Ukrainian-language texts requiring normalization to English entities;
- Multi-hop Reasoning—example: facts requiring logical inference (A inhibits B, B metabolizes C—A increases the level of C);
- Ground Truth—example: a list of triples (Source, Relation, Target) verified by an expert;

*Dataset B:* Clinical Safety QA (7_qa_dataset.json). Size: 30 test cases, 4 categories, 15 biomarkers. This dataset simulates a physician's queries to a decision support system, consisting of the following components: clinical question (question)—the physician's query; ground_truth—expert medical recommendation; contextual data: patient diagnoses (conditions), lab results (labs), drug name (drug_query), potential interactions (interaction_query). Example: a question such as “Can drug X be prescribed to a patient with condition Y?” includes numerical indicators (e.g., “Creatinine clearance < 30 mL/min”).

A purposive, criterion-based sampling strategy was used to construct the proof-of-concept corpus, with the aim of covering the principal pharmacotherapy entity types, relationships, and linguistic reasoning patterns. The exact graph size was neither predefined nor optimized against the experimental results. The final graph contained 39 nodes (13 Drugs, 1 Substance, 9 Biomarkers, 2 Targets, and 14 Conditions) and 37 directed relationships.

To reduce unsupported facts, the system used schema-constrained extraction, structured validation, and a Critic Agent assigning ACCEPT, REWRITE, or REJECT actions. Rejected or insufficiently supported facts were routed to quarantine. A confidence score, source identifier, and available supporting text were retained for each relationship. The recommendation layer applied laboratory gating and a soft ranking penalty for CONTRAINDICATED_WITH relationships; absolute drug exclusion was not implemented in the current version.

The candidate drug score was calculated as the sum of the confidence scores of supporting relationships minus the summed contraindication confidence scores multiplied by *α* = 2.0(Safety-Adjusted Base Score formula). This heuristic value assigned a contraindication twice the weight of a supporting relationship with the same confidence score. For example, a supporting relationship with a confidence score of 1.0 contributed +1, whereas a contraindication with the same confidence contributed −2. This mechanism constituted a soft ranking penalty rather than an absolute drug-exclusion rule.

End-to-end query latency was measured to assess runtime performance. Wall-clock timing started immediately before retrieval and ended after answer generation was completed. Latency was measured separately for vector RAG and GraphRAG and included retrieval and generation using Gemini 3 Flash. Offline graph construction, initial indexing, and GPT-4o/DeepEval assessment were excluded. Thirty questions were processed across three runs, providing 90 measurements per method. Latency was summarized using the mean, standard deviation, and median.

### 2.2 Experimental design: medical fact extraction (baseline, multi-agent system), comparison of RAG and GraphRAG

Five LLMs were selected for the study, divided into three strategic groups: flagship, efficient, and private (local) (Table 1).

**Table 1:** The LLMs under study and their classification into strategic groups.

Category	Model	Provider	Role in the study	Temperature parameter
flagship	GPT-5.2-Pro	OpenAI	Benchmark for top quality	Temp=0.0
flagship	Gemini 3 Pro	Google	Benchmark for long-context tasks	Temp=1.0^a^
efficient	GPT-5-Mini	OpenAI	Basic level (cost optimization)	Temp=0.0
efficient	Gemini 3 Flash	Google	High throughput (low cost)	Temp=1.0^a^
private	Llama-3.3-70B	Meta (Groq)	Open source (ability to deploy on own infrastructure)	Temp=0.0

a Note on temperature parameters: To ensure a fair comparison and prevent the degradation of reasoning specific to the Gemini 3 architecture, we set temperature = 1.0 for Google models in accordance with technical recommendations, while keeping temperature = 0.0 for OpenAI and Llama models to maximize determinism.

Medical fact extraction. The Baseline methodology (Direct Zero-Shot Extraction) was based on a Zero-Shot strategy for direct knowledge extraction in a single pass, without the use of agent loops or external verification tools (run_exp_direct_llm.py). The process was implemented via a pipeline (chain) that includes a prompt template, an LLM, and a structured output parser. The model assumes the role of a medical data expert. Key instructions included cross-lingual processing (e.g., translation of medical terminology from Ukrainian to English), the use of standard relation types (TREATS, INHIBITS, etc.), and strict adherence to numerical constraints (e.g., eGFR < 30). Experiments were conducted with fixed parameters: temperature = 0.0(1.0) and seed = 42. The integrity of the input data was verified using the SHA-256 hash of the dataset file. To calculate metrics, the results were processed through two types of normalizers implemented in the TripletNormalizer class: Strict Normalizer—performs minimal processing (converting to lowercase and removing extra spaces) while preserving punctuation and suffixes. Relaxed Normalizer (1:1)—an algorithm that removed punctuation, truncated contextual suffixes (e.g., “levels,” “virus”), used synonym mapping, and parsed numerical constraints to compare their mathematical essence rather than just the text.

The agent methodology (Multi-Agent System) is implemented as an iterative conveyor based on LangGraph, which transforms the extraction process into a system with three processing nodes and a built-in self-correction mechanism. The system is controlled by the GraphState object, which ensures end-to-end context transfer between nodes.

Extractor Node: performs initial extraction. If errors occur in the JSON structure, the OutputFixingParser is automatically triggered.

Ontology Node: performs entity normalization (the MVP version functions as a pass-through node for subsequent integration with UMLS/RxNorm).

Critic Node (LLM-as-a-Judge): each fact is evaluated based on clinical safety and confidence criteria.

Extraction metrics. Metrics are used to quantitatively assess the accuracy of triplet extraction by comparing the set of predicted facts P with the reference facts E.

Definition of “Strict Match”. Before counting the numbers, the system compares two lists of facts:

- Expected (Ground Truth): reference facts from Dataset A;
- Predicted: facts generated by the model.

In STRICT mode, a fact is considered found (True Positive) if and only if all three components of the triplet match:

$$\left(\mathrm{Subjec}t_{\mathrm{pred}}==\mathrm{Subjec}t_{\mathrm{ref}})\wedge (\mathrm{Relatio}n_{\mathrm{pred}}==\mathrm{Relatio}n_{\mathrm{ref}})\wedge (\mathrm{Objec}t_{\mathrm{pred}}==\mathrm{Objec}t_{\mathrm{ref}}\right)$$

True Positives (TP): The number of predicted triples that match the benchmark according to the selected matching algorithm (Strict or Relaxed).

False Positives (FP): The number of extracted triples that are not present in the benchmark dataset (hallucinations).

False Negatives (FN): The number of reference triples that the model failed to extract.

Precision (Strict Precision, strict_graph_precision): The proportion of correctly identified facts among all those found by the model.

$$\mathrm{Precision}=\frac{TP}{TP+FP}$$

Recall (Strict Recall, strict_graph_recall): The proportion of facts found relative to the total number of facts contained in the text.

$$\mathrm{Recall}=\frac{TP}{TP+FN}$$

The harmonic mean (Strict F1-Score, strict_graph_f1) between precision and recall, which provides a balanced evaluation.

$$F_{1}=2\cdot \frac{\mathrm{Precision}\cdot \mathrm{Recall}}{\mathrm{Precision}+\mathrm{Recall}}$$

False Positive Count (FP Count, strict_fp_count). The absolute number of irrelevant facts.

### 2.3 Methodology for the statistical analysis of the medical data extraction experiment

To evaluate the effectiveness of the Multi-Agent System methodology and the Baseline approach, we applied a two-level statistical approach that accounts for the hierarchical structure of the experimental data (crossover design: 12 cases × 5 models).

Linear mixed-effects model (LMM) was used. The Strict F1 Score was the dependent variable. This model also enabled us to estimate the effect of the methodology (β_1), whilst controlling for the variance of the models' baseline performance and the heterogeneity of clinical scenarios.

The model was characterized as follows:

$$y_{ijk}=\beta_{0}+\beta_{1}\cdot Method_{i}+u_{j}+v_{k}+\int_{ijk}$$

where:

- $y_{ijk}$ is the metric value (Strict F1, Strict Precision, Strict Recall) for the i-th observation;
- $\beta_{0}$ represents the average performance of all baseline models (excluding random fluctuations);
- $\beta_{1}$ is the fixed-effect coefficient (Multi-Agent System);
- $\mathrm{Metho}d_{i}\;\;$—a binary variable (1 for Multi-Agent System and 0 for Baseline);
- $u_{j}∼N(0,\sigma_{u}^{2})$—a random intercept for the *i*-th clinical case (CaseID), accounting for the heterogeneity (varying complexity) of tasks;
- - $v_{k}∼N(0,\sigma_{v}^{2})\;\;$—a random intercept for the *k*-th model provider (Provider), accounting for differences in the models' inherent capabilities;
- $\epsilon_{ijk}$—residual error.

Robustness Check. Given the relatively small number of clinical cases (*n* = 12) included in the study, we additionally performed a nonparametric permutation sign-flip test. For each iteration, the sign of the delta (Δ = Agentic—Base) was randomly flipped to its opposite to generate a null distribution and calculate an empirical *p*-value. This method served as a conservative check to determine whether the overall success of the experimental methodologies was due to outliers in the sample.

Multiple Comparisons Adjustment. For all secondary comparisons, across categories of pharmacological knowledge, the Holm-Bonferroni sequential correction procedure was applied to strictly control the group-wise Type I error rate (FWER). Statistical calculations were performed using the statsmodels and scipy libraries in Python. The significance level for rejecting the null hypothesis was set at *α* = 0.05.

To compare the performance of traditional vector search (RAG) and the proposed graph-oriented approach (GraphRAG), a testing system based on the DeepEval framework (v3.7.5) was used. The reproducibility of the results was ensured by fixing the seed values of the pseudorandom number generators and saving the hashes of the input data (SHA-256). The evaluation was performed on Dataset B (run_exp_graph_vs_rag.py). The knowledge base included medical documents (documents table), a knowledge graph with nodes (nodes table: drugs, diseases, biomarkers) and edges (edges table: relationships such as TREATS, CONTRAINDICATED_WITH, INCREASES, DECREASES) with confidence_score and properties attributes. Total number of cases: 30.

Classic RAG algorithm. Documents from the database were vectorized using a model (text-embedding-3-small) and indexed in the Chroma vector store. For each query, a cosine-similarity search was performed, returning k = 3 of the most relevant documents. The LLM generated a response based on a prompt that included the retrieved documents as context and a clinical question. A two-model approach was used: lm_doctor (doctor model) and llm_evaluator (judge model).

The GraphRAG algorithm provides:

- hybrid entity search via exact matching (ILIKE) to find nodes by name; if no match is found, vector search with a cosine distance threshold of ≤0.8; classification of biomarkers via BiomarkerRegistry (reference ranges, critical thresholds);
- extraction of TREATS relationships—direct drug-disease relationships, alternative drugs for the same disease (top 5 by confidence_score), and other clinically significant relationships between a drug and a diagnosis;
- analysis of contraindications, CONTRAINDICATED_WITH links from the edges table (bidirectional search), low-confidence contraindications from the quarantine table (status: under_review), application of a penalty factor: penalty = 2.0 × confidence_score;
- laboratory interactions, extraction of INCREASES/DECREASES relationships for biomarkers, classification of patient status: HIGH/LOW/NORMAL relative to reference ranges;
- interactions with active pharmaceutical ingredients, checking INTERACTS_WITH relationships for queries;
- search for alternatives—drugs with TREATS/DECREASES relationships to target entities (contraindications, elevated biomarkers);
- output documents—download of full-text source documents by source_doc_id.

For the RAG and GraphRAG methodologies, the doctor model generated a response (actual_output), while the judge model evaluated the correctness of the generated responses by comparing them to the ground truth (expected_output) and assigning scores between 0 and 1, as well as using the DeepEval metric (Medical Accuracy).

### 2.4 Statistical analysis of RAG and GraphRAG results

For each model configuration, multiple runs (num_runs) were performed with incremental seed values [seed, seed+1, … , seed + *n* − 1]. This allowed for an assessment of the variability in results due to the stochasticity of the LLM. For each clinical case, paired differences *δ*ᵢ = Medical_Accuracy_GraphRAG—Medical_Accuracy_RAG were calculated:

1. Wilcoxon signed-rank test (non-parametric, two-tailed)—applied when there were ≥6 non-zero paired differences, testing the hypothesis H₀: median *δ* = 0;
2. Paired *t*-test (parametric, two-tailed)—applied when *n* ≥ 2 cases, testing the null hypothesis H₀: E[*δ*] = 0;
3. Effect size (Cohen's d): d = mean(*δ*)/std(*δ*). |d| ≥ 0.8 (large), ≥0.5 (medium), ≥0.2 (small), <0.2 (negligible).

Significance level: *α* = 0.05 for all statistical tests.

Win-rate analysis: the number of cases where GraphRAG_wins (*δ* > 0), RAG_wins (*δ* < 0), and ties (*δ* = 0) was counted. Given that LLMs are probabilistic by nature, the results of a single experiment do not prove the superiority of the architecture. Multiple runs allow us to account for the randomness factor. This metric answers the question: “In what percentage of cases will a doctor get a better answer using GraphRAG?”

## 3 Results

The implementation of the agent-based architecture led to improvements in all strict precision metrics (F1, Precision, Recall) (Table 2). The most significant recall improvement (0.172, *p* < 0.001) was obtained by the standard extraction of medical-pharmacological relations, confirming the usefulness of the coordination among agents to avoid information loss.

**Table 2:** Comparison results of the baseline zero-shot and multi-agent system methodologies.

Metric	Baseline (Mean)	Agentic (Mean)	Diff (Coef)	SE	*p*-value (one-sided)	Mixed-Effects Coef (*β*)	*p*-value (Primary)	Permutation p (Robustness)
Strict Graph F1	0.4417	0.5975	+0.1559	0.0552	0.0024**	+0.1559	0.0024**	0.1639 (ns)
Strict Precision	0.4556	0.6110	+0.1555	0.0561	0.0028**	+0.1555	0.0028**	0.1798 (ns)
Strict Recall	0.4389	0.6111	+0.1722	0.0546	0.0008***	+0.1722	0.0008***	0.1121 (ns)

Significance levels:

*p* < 0.05.

** *p* < 0.01.

*** *p* < 0.001.

The nonparametric permutation test (Robustness Check) showed a consistent direction of change (positive delta); however, due to the limited sample size (*n* = 12), the obtained *p*-values (0.11–0.18) did not cross the conservative significance threshold.

**Figure 2:**
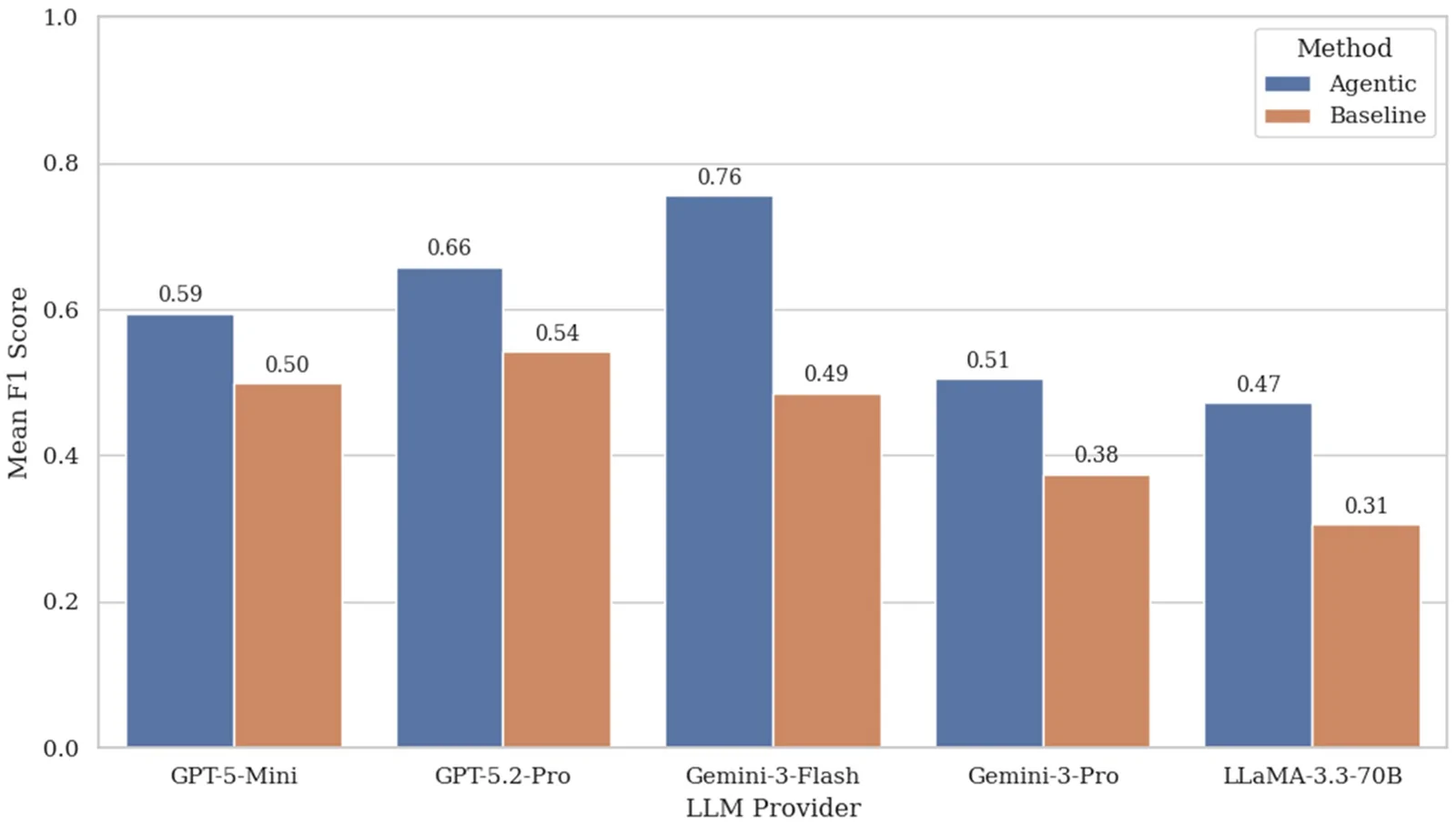
Strict F1 distribution according to LLM provider.

As shown in Figure 2, the implementation of the agent-based architecture ensured a stable increase in the Strict F1 metric for all five tested LLMs, regardless of their size (number of parameters) or provider. This confirms the hypothesis regarding the universality of the proposed method.

The Gemini-3-Flash model demonstrated the most significant results. In baseline mode, its performance was F1 = 0.49, but in agent mode, it reached the absolute maximum among all models—0.76. This result even surpassed the flagship GPT-5.2-Pro model (F1 = 0.66 in agent mode). For the LLaMA-3.3-70B model, the agent approach yielded an increase from 0.31 to 0.47. Although it still lags behind proprietary models, this leap takes it from the “unsuitable” level to a level comparable to the baseline performance of GPT-5-Mini (0.50). In Baseline mode, the spread of results among models was significant (from 0.31 to 0.54). In Agent mode, productivity stabilized, and the lower bound rose to 0.47.

**Figure 3:**
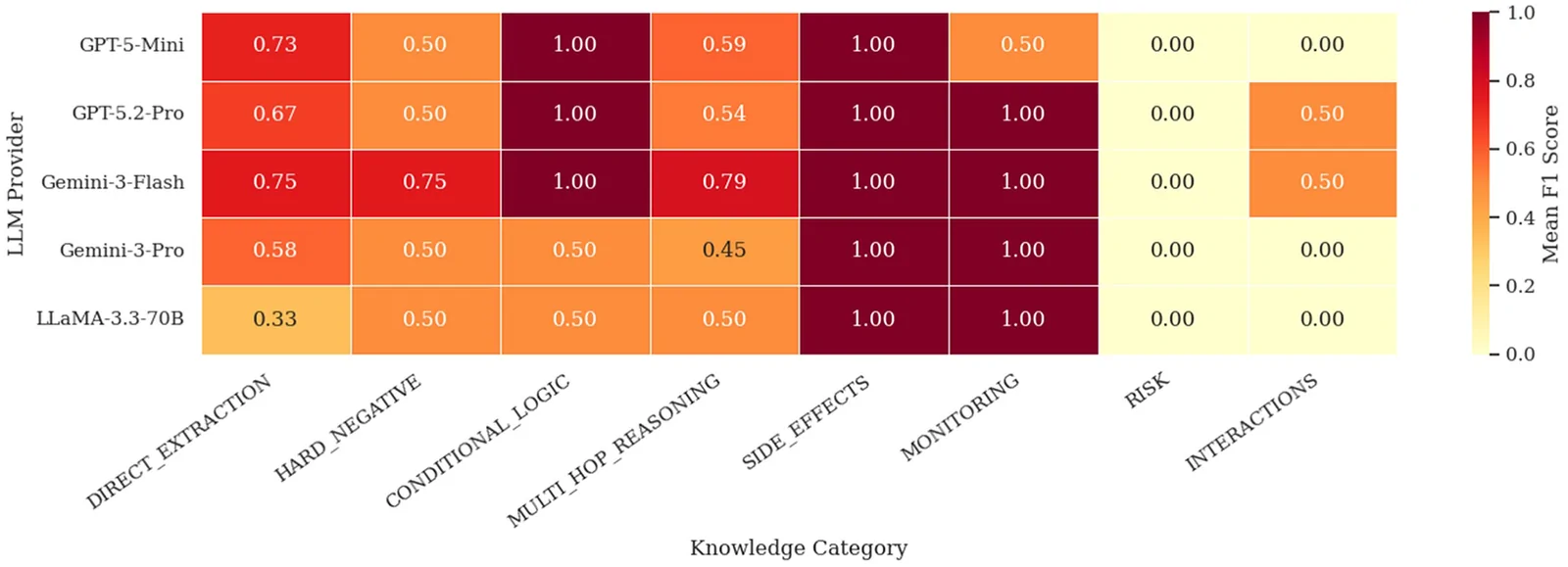
Heatmap of the performance (strict F1) of the agent-based method across clinical knowledge categories. The variation in results (Mean F1 Score) for five LLMs is shown. Dark red cells indicate perfect extraction (F1 ≈ 1.0), while light (yellow) cells show areas where the models failed to identify correct relationships (F1 ≈0.0).

A detailed analysis by knowledge category (Figure 3) highlights the uneven distribution of efficiency depending on the task type, allowing clinical knowledge categories to be classified into three levels of complexity.

High performance. Categories based on direct linguistic markers, such as “Side Effects” and “Monitoring,” were identified nearly without error. Most models achieved an F1 = 1.0 score here, indicating the ability of the agent system to effectively filter explicit facts from the text, regardless of the base model;

Variable performance in logical tasks. In categories requiring information synthesis, significant differentiation is observed. In the Conditional Logic category, the Gemini-3-Flash model demonstrated unprecedented accuracy (F1 = 1.0), while other models, including LLaMA-3.3, showed mixed results (0.5). In the Multi-Hop Reasoning category, Gemini-3-Flash (F1 = 0.79) significantly outperformed GPT-5.2-Pro (F1 = 0.54) in its ability to maintain context across long chains of dependencies;

Low performance. The “Risk” and “Interactions” categories proved to be the most difficult. For most models, the F1 score here approached 0.0. This “failure” indicates a certain limitation of the architectures in detecting implicit causal relationships, which are often not explicitly stated in the text but require deep medical background knowledge.

A comparative study was conducted between traditional vector search (RAG) and the proposed graph-oriented approach (GraphRAG). Given that the Gemini-3-Flash model proved promising in previous results, it was used as the “doctor” model, while the classic GPT-4O model was used as the “judge” model to ensure objective evaluation.

**Figure 4:**
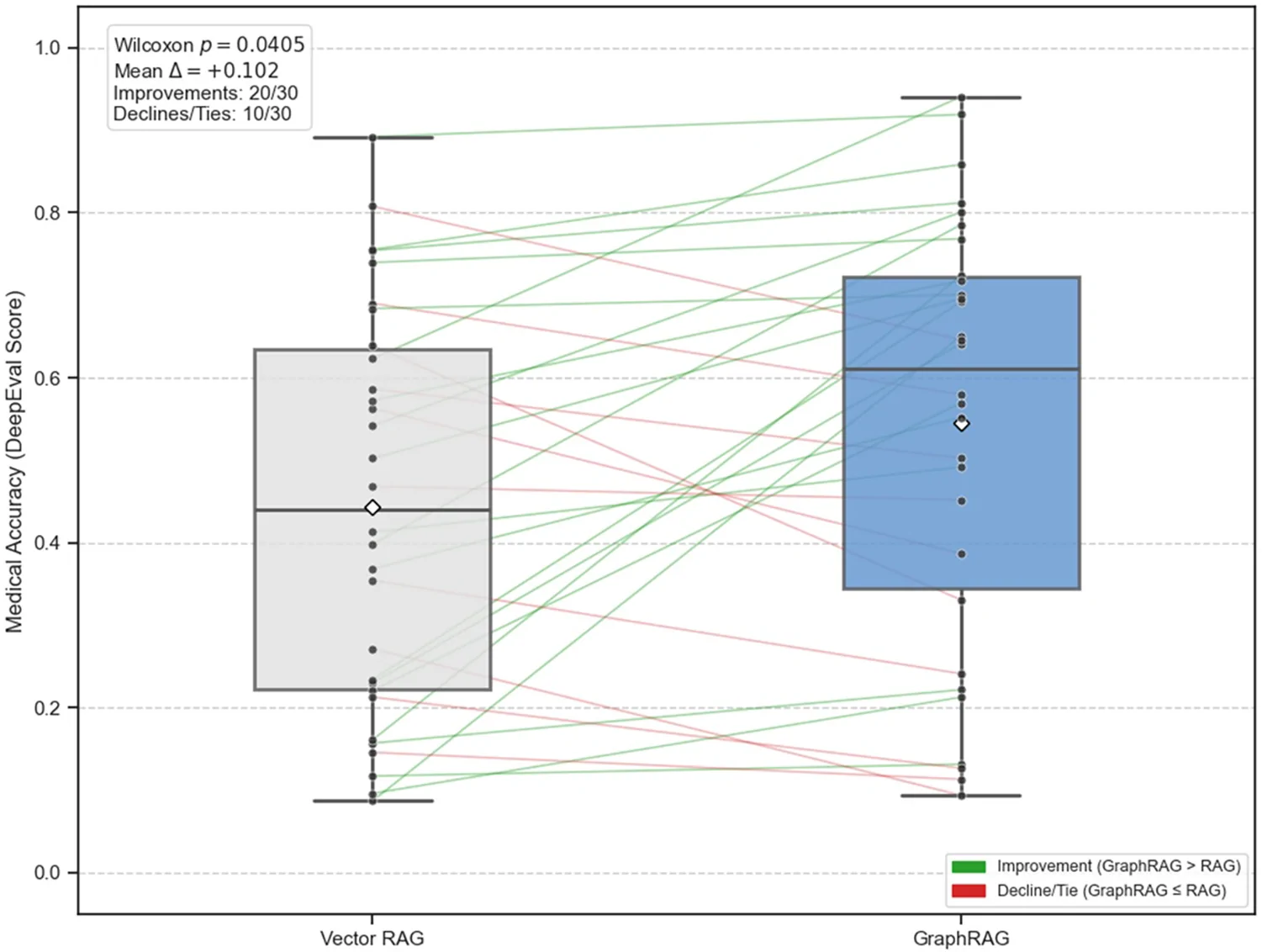
Paired performance analysis of the vector RAG and GraphRAG methods (*n* = 30). The box plot displays the distribution of the Medical Accuracy metric for 30 clinical cases. The connecting lines show the individual change in accuracy for each case: green lines indicate an improvement in results when using GraphRAG, while red lines indicate a deterioration or no change. Statistical significance was confirmed by the Wilcoxon test (*p* = 0.04).

The aggregated results of three independent experimental runs (3 runs × 30 cases) demonstrate the superiority of the graph-oriented approach over traditional vector search (Figure 4).

The average accuracy (Medical Accuracy) for GraphRAG was 0.545 ± 0.258, whereas for the baseline RAG this parameter was 0.443 ± 0.243 (Table 3). The average accuracy gain (Delta, *Δ*) when using GraphRAG was +0.102 (or +10.24% in absolute accuracy). It is important to note the stability of this gain across all three runs with different random number generator seed values:

Run1:Δ=+0.0897;Run2:Δ=+0.1138;Run3:Δ=+0.1037.

**Table 3:** Statistical comparison metrics for vector RAG and GraphRAG.

Test	Statistics	*p*-value	Indicator	RAG	GraphRAG
Wilcoxon signed-rank	W = 133.0	*p* = 0.040	Mean accuracy	0.443 ± 0.243	0.545 ± 0.258
Paired *t*-test	*t* = 2.47	*p* = 0.020			
Cohen's d	0.451	small-to-medium			

This indicates that the improvement is a result of GraphRAG's architectural advantages, rather than random fluctuations inherent in the stochastic nature of LLMs.

Statistical analysis confirmed the significance of the results at the *α* = 0.05 level. Wilcoxon signed-rank test: W = 133.0, *p* = 0.040. Since *p* < 0.05, the null hypothesis of no difference in medians is rejected. Paired *t*-test: *t* = 2.47, *p* = 0.019. Since *p* < 0.05, the difference in means is statistically significant. The effect size (Cohen's d) was 0.451, which can be interpreted as a medium effect size. In our opinion, this indicates that switching to GraphRAG results in a noticeable improvement in the quality of responses in practice.

Win-Rate analysis and error topology. GraphRAG outperformed RAG in 20 out of 30 cases (66.7%), while RAG performed better in 10 cases. The largest increase, Δ > +0.4, was observed in the SAFETY (e.g., case EN_20) and NUMERIC_RISK (case EN_07) categories. In these cases, GraphRAG successfully extracted hard constraints from graph edges (CONTRAINDICATED_WITH) and numerical values from the biomarker registry, which vector search “blurred” in context. An analysis of 10 cases where RAG outperformed the baseline revealed structural limitations of the current graph: absence of an entity in the query (Null Query): in cases EN_24 and EN_29, the algorithm failed to identify the key entity (drug name), resulting in an empty graph context. Complex mechanisms of action: RAG found a textual description of the mechanism in case of EN_22 (Resveratrol + mTOR), but the graph did not have a “mTOR” node or the link between the two, which leads to a loss of context.

GraphRAG did not demonstrate a noticeable increase in response time compared with vector RAG. Mean end-to-end latency was 5.902 ± 3.478 s for GraphRAG and 6.192 ± 4.291 s for vector RAG. Median latency was 4.425 and 4.593 s, respectively. The descriptive mean difference was −0.290 s in favour of GraphRAG (Table 4). Given the substantial between-run variability of the external API, these results were not interpreted as evidence that GraphRAG was statistically faster.

**Table 4:** End-to-end query latency for vector RAG and GraphRAG.

Execution set	Measurements per method	Vector RAG, mean ± SD (s)	GraphRAG, mean ± SD (s)	*Δ* GraphRAG—RAG (s)
Run 1, seed 42	30	6.644 ± 4.726	5.447 ± 3.271	−1.197
Run 2, seed 43	30	5.510 ± 3.827	6.120 ± 3.293	+0.610
Run 3, seed 44	30	6.422 ± 4.335	6.139 ± 3.906	−0.283
All executions	**90**	**6.192** ± **4.291**	**5.902** ± **3.478**	**−0**.**290**

Latency included retrieval and answer generation but excluded offline graph construction, initial indexing, and GPT-4o/DeepEval assessment. The 90 measurements represent 30 questions repeated across three runs rather than 90 independent clinical cases. Negative *Δ* values indicate lower descriptive latency for GraphRAG. Bold values indicate the aggregate results across all three runs.

## 4 Discussion

The main analysis (LMM) indicated highly statistically significant results (*p* < 0.01), but the conservative permutation test did not reach significance at *α* = 0.05. This difference is a methodological artefact that one would expect when working with small sample sizes (*n* = 12). The permutation test is “flat” with respect to the data, and does not take into account the internal variance due to different complexity of the clinical cases. Instead it explicitly models this heterogeneity by the inclusion of random effects (1|CaseID). We argue that the LMM results are more representative of the real impact of the architecture, since the LMM is better at separating the signal from the noise. Moreover, from a clinical perspective, an effect size exceeding 15% (notably for Strict Recall) is very relevant for decision support systems where the cost of an error (a missed drug interaction) is far greater than the risk of statistical uncertainty.

Our results cast doubt on the assumption that only expensive flagship models (State of the Art, SOTA) are necessary for high-precision clinical extraction. The success of Gemini-3-Flash (Figure 2), which outperformed the significantly more expensive GPT-5.2-Pro in agent mode, demonstrates the “architectural compensation” effect. A specialized multi-agent system (Extractor + Critic) is capable of compensating for a smaller number of model parameters, correcting its hallucinations and logical errors. This paves the way for cost-effective deployment. Hospitals can use cheaper and faster models (such as Flash or Mini) without sacrificing quality, achieving accuracy that was previously only available when using the most expensive LLMs.

The significant performance gain of LLaMA-3.3 (+51%) points to the potential for creating fully autonomous (on-premise) systems that operate locally without transferring patient data to the cloud. Although open-source models still require refinement to reach the level of GPT-5, the agent-based architecture makes them viable candidates for tasks with heightened data privacy requirements (GDPR/HIPAA compliance).

The dichotomy we found between high accuracy in “Side Effects” and low accuracy in “Interactions” suggests that agent-based architecture solves the attention problem but not the domain knowledge gap.

Despite the advantages of agent-based architecture, a detailed analysis of instances where the system made errors revealed specific patterns characteristic of multi-agent systems. In isolated cases, the planner agent generated requests to non-existent graph nodes or interpreted an intermediate response from one agent as a confirmed fact without waiting for verification from the validator agent. This led to the formation of logically structured but factually incorrect relationships. When analyzing particularly complex medical cases (for example, in the INTERACTIONS categories), agents sometimes entered a loop of verifying the same fact through various intermediate arguments. This did not result in a runtime error, but it exhausted the token limit, leading to a response cutoff and a decrease in recall. Agents sometimes attempted to break down simple medical facts into overly small atomic relationships that did not correspond to the “ground truth”. This explains the slight underperformance of agents in the DIRECT_EXTRACTION category, where the direct response of the model was closer to the target graph than a multi-step inference.

In our opinion, an important metric of this study in the field of medical data is the use of Strict-Graph F1 instead of traditional text similarity evaluation methods, such as ROUGE or BLEU, which is consistent with other studies (36–38).

Traditional metrics (BLEU/ROUGE) are based on word overlap (n-grams) which is not acceptable in medicine, where changing one term or negation changes the clinical meaning completely. While Strict-Graph F1 requires the exact match of the whole subject-predicate-object relationship triad. The metric we use does not account for partial matches. If the agent correctly identified the drug but made a mistake regarding the type of its interaction (for example, stating “treats” instead of “exacerbates the side effect”), such a result is scored as 0. This gives the results high weight, as they reflect the system's actual readiness for use in clinical practice, where the cost of an error is critical. Using a graph-based approach allows us to mitigate the impact of the “verbosity” of models by focusing exclusively on structured knowledge (39). This makes our comparison of models with different generation styles (e.g., the concise Gemini and the verbose GPT) as unbiased as possible.

Our results confirm and extend the findings of important studies in the field of medical AI and agent-based systems, while offering advantages in data structuring accuracy. For instance, the ReAct methodology (40) demonstrated that combining logical inference and action execution improves performance in knowledge-based tasks. However, unlike the classic ReAct, our approach integrates graph verification at every stage. While ReAct may suffer from “reasoning drift,” where an error in the second step breaks the entire chain, our agent-based graph-oriented approach ensures structural fixation of facts. This has allowed us to achieve higher performance in the Conditional Logic category, where standard chains of reasoning (CoT) often lose logical integrity.

Research on Med-PaLM 2 has shown that models with 540 billion parameters can achieve expert-level performance on medical exams (40). However, our experiment with Gemini-3-Flash demonstrates that competitive accuracy (F1 0.756) can be achieved on significantly more compact models using an agent-based architecture and GraphRAG. Our approach solves the main problem of Med-PaLM 2—the difficulty of extracting structured relationships for medical databases—since Med-PaLM 2 is optimized for text-based answers, whereas our system generates ready-to-use graph structures.

Current implementations of GraphRAG (Microsoft Research) focus on global document understanding (41). Our work adapts this idea for microstructural accuracy in medicine. Instead of a general description of entities, our approach uses agents to strictly verify each predicate (e.g., differentiating between “inhibition” and “antagonism”), as reflected in the use of the Strict-Graph F1 metric.

## 5 Conclusion

It has been established that a specialized agent architecture is capable of compensating for a smaller number of model parameters. The use of the cost-effective Gemini-3-Flash model in multi-agent mode (F1 = 0.76) allowed it to statistically significantly outperform the results of the significantly more expensive flagship GPT-5.2-Pro model (F1 = 0.66). This paves the way for its cost-effective implementation in healthcare facilities.

The GraphRAG approach led to a considerable increase in medical accuracy over the conventional vector search (Δ = +0.102, *p* = 0.04). This advantage was especially crucial in the areas of safety and risk analysis, where the graph structure guarantees that stringent requirements (e.g., contraindications) are considered, preventing the loss of important information which is typical of “fuzzy” semantic search.

Unlike classical methodologies such as ReAct or standard chains of reasoning (CoT), the implementation of graph verification at every stage of the agents' operation prevents the breakdown of logical integrity. This is confirmed by high results (F1 = 1.0) in conditional logic tasks.

The observed performance gain of open-source models (e.g., LLaMA-3.3 by +51%) confirms the fundamental feasibility of creating autonomous (on-premise) medical decision support systems, which is a critically important step for complying with strict patient data privacy requirements (GDPR/HIPAA).

## 6 Study limitations

### 6.1 Known limitations

Despite the high statistical significance and practical value of the obtained results, this study has a number of limitations that must be taken into account when interpreting the data and planning future research.

### 6.2 Sample size limitation

Experiments were performed on relatively small datasets (*n* = 12 for architecture comparisons, *n* = 30 for RAG/GraphRAG comparisons). The linear mixed model (LMM) was significant (*p* < 0.01) but did not reach the threshold of *α* = 0.05 in a conservative nonparametric permutation test. A definitive generalisation of the results needs validation in larger clinical cohorts.

### 6.3 The problem of structural rigidity (rigidity of GraphRAG)

The graph-oriented approach showed high sensitivity to the completeness of the ontology. In cases where the target entity was absent from the query (Null Query) or a specific node was missing from the graph (e.g., the molecular target “mTOR”), GraphRAG returned an empty context. In such situations, the traditional vector-based RAG demonstrated greater flexibility by relying on semantic similarity of the text.

### 6.4 Lack of domain knowledge in implicit tasks

The system demonstrated a critical decrease in efficiency (Strict F1 = 0.0) in the “Risk” and “Interactions” categories.

The agent-based architecture solves the problem of attention concentration, but in Zero-Shot mode, it is unable to compensate for the lack of deep medical knowledge required to infer implicit causal relationships.

### 6.5 Hyper-segmentation of facts

In simple tasks (Direct Extraction), agents exhibited a tendency to excessively break down information into atomic relationships, which did not always correspond to the ground truth and resulted in a slight penalty on the Strict-Graph F1 metric compared to the direct answers of baseline models.

The system was not evaluated using real EHR data; therefore, its clinical performance and generalizability remain unknown. The next validation stage should include retrospective evaluation using de-identified electronic health records, followed by prospective silent-mode validation. The Ukrainian digital health ecosystem is considered the initial implementation context, subject to the technical capabilities and permitted data flows available through the medical information system and the central database of the Ukrainian Electronic Health Care System (ЕСОЗ).

## Directions for further research

In future studies, it would be advisable to expand testing of the approach to specific medical domains.

## Data Availability

The raw data supporting the conclusions of this article will be made available by the authors, without undue reservation.
